# The Development of Serum Amyloid P as a Possible Therapeutic

**DOI:** 10.3389/fimmu.2018.02328

**Published:** 2018-10-16

**Authors:** Darrell Pilling, Richard H. Gomer

**Affiliations:** Department of Biology, Texas A&M University, College Station, TX, United States

**Keywords:** pentraxin, serum amyloid P component (SAP), fibrosis, macrophage, fibrocyte, pulmonary fbrosis

## Abstract

Pentraxins such as serum amyloid P (SAP; also known as PTX2) regulate several aspects of the innate immune system. SAP inhibits the differentiation of monocyte-derived fibroblast-like cells called fibrocytes, promotes the formation of immuno-regulatory macrophages, and inhibits neutrophil adhesion to extracellular matrix proteins. In this minireview, we describe how these effects of SAP have led to its possible use as a therapeutic, and how modulating SAP effects might be used for other therapeutics. Fibrosing diseases such as pulmonary fibrosis, cardiac fibrosis, liver fibrosis, and renal fibrosis are associated with 30–45% of deaths in the US. Fibrosis involves both fibrocyte differentiation and profibrotic macrophage differentiation, and possibly because SAP inhibits both of these processes, in 9 different animal models, SAP inhibited fibrosis. In Phase 1B and Phase 2 clinical trials, SAP injections reduced the decline in lung function in pulmonary fibrosis patients, and in a small Phase 2 trial SAP injections reduced fibrosis in myelofibrosis patients. Acute respiratory distress syndrome/ acute lung injury (ARDS/ALI) involves the accumulation of neutrophils in the lungs, and possibly because SAP inhibits neutrophil adhesion, SAP injections reduced the severity of ARDS in an animal model. Conversely, depleting SAP is a potential therapeutic for amyloidosis, topically removing SAP from wound fluid speeds wound healing in animal models, and blocking SAP binding to one of its receptors makes cultured macrophages more aggressive toward tuberculosis bacteria. These results suggest that modulating pentraxin signaling might be useful for a variety of diseases.

## Introduction: SAP and debris clearance

**Figure 1:**
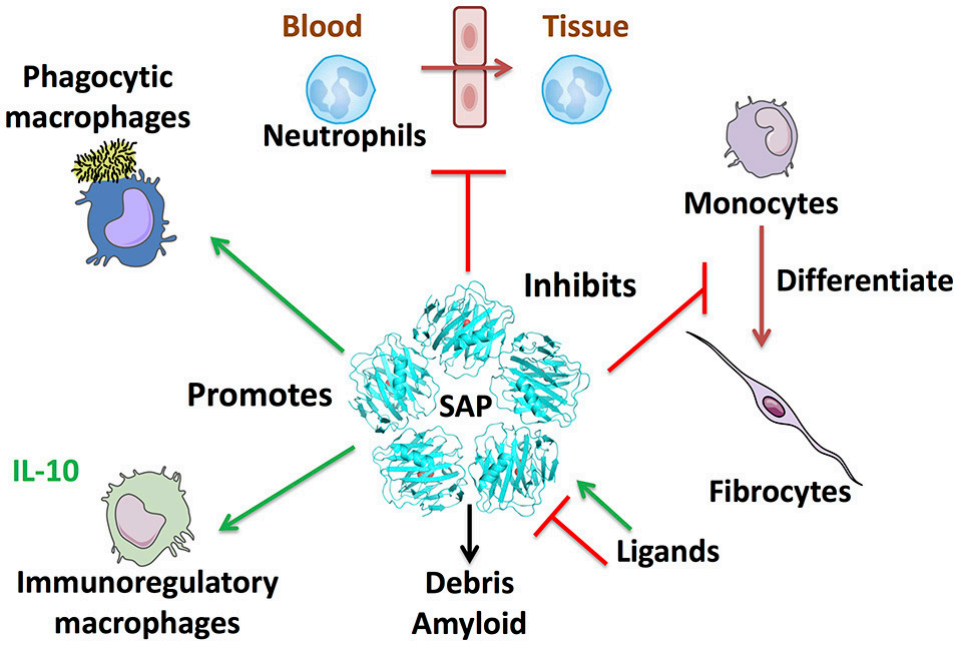
SAP regulates multiple aspects of immune responses. Some of the known effects of SAP are shown clockwise from top: SAP inhibits neutrophil adhesion to extracellular matrix and inhibits neutrophil movement into tissues. SAP binds to FcγR and DC-SIGN to inhibit monocyte to fibrocyte differentiation. SAP also binds multiple plasma proteins such as the complement component C1q and mannose-binding lectin (MBL) to promote phagocytosis of bacteria and regulate macrophage differentiation. SAP opsonizes bacteria and cell debris to promote removal by macrophages, and binds amyloid deposits. Finally, SAP promotes immuno-regulatory, and M1 phagocytic macrophages.

SAP (PTX2) is a member of the pentraxin family of proteins that includes C-reactive protein (CRP; PTX1) and pentraxin-3 (PTX3). SAP is made by hepatocytes and secreted into the blood (1, 2). Searches of proteomics and RNA-seq databases suggests that the liver is the major source of SAP. In humans and most mammals, the levels of SAP in the plasma are maintained at relatively constant levels, between 20 and 50 μg/ml (3–5). There is little evidence for sequence variation of SAP at the genomic or amino acid level. In mice, SAP acts as an acute phase protein, with levels rising up to 20-fold following an inflammatory insult (6, 7). SAP is a pentameric protein with sequence and structural similarity to CRP (8–10). The structure of SAP (and CRP) pentamers is a flat disk with a hole in the middle (11, 12). The crystal structure of PTX3 has yet to be determined, but models based on site-directed mutagenesis, electron microscopy, and small-angle X-ray scattering data suggests that PTX3 is an octamer of two tetramers (13). Each SAP molecule has two Ca^++^ atoms bound to it, and the pentamer thus has 10 Ca^++^ atoms on one side of the disk. With the help of the bound Ca^++^, this side of the disk binds to a variety of molecules including apoptotic debris, bacterial polysaccharides, amyloid deposits, and bacterial toxins (1, 14, 15). Phagocytic cells such as monocytes and macrophages then bind the SAP, CRP, or PTX3, and engulf the debris or other material the pentraxin has bound (16). CRP and PTX3 can similarly bind a variety of debris molecules (17, 18). Proteins with strong similarity to SAP (and CRP and PTX3) are present in the hemolymph of horseshoe crabs (19, 17), so this debris clearance mechanism appears to have evolved during the early evolution of animals (Figure 1).

## Removing stuck SAP as a possible therapeutic for amyloidosis

Amyloidosis is a disease where misfolded proteins aggregate and form large deposits in a tissue, leading to organ dysfunction (20, 21). SAP was originally isolated as a serum-derived protein found in all types of amyloid deposits (hence the serum amyloid part of its name) (22, 23). SAP was found to be a pentameric protein, hence the P part of its name (24–27). SAP is also easily purified by incubating serum with certain types of agarose in the presence of calcium, washing unbound protein off, and then eluting fairly pure bound SAP with a calcium chelator (28, 29). One possibility is that the SAP in the amyloid deposits binds to the misfolded proteins in an attempt to opsonize them for phagocytosis, but cannot pull proteins out of the deposit, and the SAP then gets stuck in the deposits. SAP knockout mice have reduced severity of experimentally-induced amyloidosis, suggesting that the stuck SAP exacerbates the amyloid deposit formation and/or hinders the ability of other opsins to pull the amyloid complexes apart (30). The Pepys group found a small molecule compound that causes two human SAP pentamers to stick to each other, and this complex is then quickly cleared from the circulation (31). In SAP knockout mice expressing human SAP, the compound decreased serum SAP levels but did not reduce the severity of experimentally-induced amyloidosis (31). Adding anti-SAP antibodies to this treatment however did reduce experimentally-induced amyloidosis, suggesting that reducing SAP levels is a possible therapeutic for amyloidosis (32–35).

## Intermezzo 1: wound healing and fibrosis

Most plant and animal tissues have a remarkable ability to heal mechanical wounds, indicating a strong evolutionary pressure for wound healing (36). In vertebrates, a typical dermal wound fills with scar tissue consisting of fibroblasts, connective tissue, and a capillary bed, and then is covered with an epithelium (37). Unfortunately, inappropriate wound healing responses to perceived wounds cause fibrosing diseases, where scar tissue forms in an internal organ, leading to organ dysfunction. There are at least 62 different fibrosing diseases, and these are associated with 30–45% of deaths in the US (38, 39). Examples of fibrosing diseases include cardiac fibrosis, probably triggered by reduced blood flow to part of the heart, and this fibrosis accounts for a significant fraction of the 450,000 deaths per year from cardiovascular disease in the US (40, 41). Other fibrosing diseases are cirrhosis of the liver, triggered by damage from viral infections, alcohol, or other chemical insults (39), end-stage kidney disease in diabetics, where the scar tissue formation is probably triggered by damage from high glucose levels (42), and pulmonary fibrosis, where particulate matter such as coal dust, and other unknown factors, triggers the progressive formation of scar tissue in the lungs (43). The only FDA-approved therapeutics for fibrosis are two drugs which slow, but do not stop, the progression of pulmonary fibrosis (44).

## Intermezzo 2: fibrocytes

In the 1850's, James Paget examined healing wounds and observed that cells from the blood enter the wound and then differentiate into elongated cells with an oval nucleus [see Figure 14, page 127 in (45)]. Bucala et al. found that these cells originate from bone marrow derived circulating CD14^+^ monocytes and express markers such as such as CD34 and CD45 that identify them as bone marrow-derived cells, as well as markers such as collagen that identify them as fibroblast-like cells (46–49). They named the cells fibrocytes. Although fibrocytes are rarely observed in normal tissues, they are present in high numbers in healing wounds (46, 50, 51) and fibrotic lesions in pulmonary fibrosis (51–58), keloid scars (59, 60), asthma (52, 61, 62), chronic kidney disease (63, 64, 65), and nephrogenic systemic fibrosis (66). Fibrocytes are also present in the fibrotic lesions in animal models of pulmonary fibrosis (53, 67–75), liver fibrosis (71) and renal fibrosis (73, 76). In addition to contributing to the mass of fibrotic lesions, fibrocytes promote angiogenesis (77), which can then promote the growth of the scar tissue, and secrete TGF-β (78), which causes resident fibroblasts to proliferate and increase their collagen production. Fibroblasts thus have a multiplicative effect on scar tissue formation. At the time we started working on fibrocytes, nothing was known about extracellular factors that regulate their differentiation, and thus why fibrocytes are present in wounds but not normal tissues, and how to control them.

## Our entry into the SAP, fibrocytes, wound healing, and fibrosis fields

Our lab had been studying the ability of diffusible secreted factors to indicate the local density and composition of cells in a tissue, using the eukaryotic amoeba *Dictyostelium discoideum* as a model system (79, 80). We decided to try to look for cell density sensing factors secreted by human white blood cells. To simplify the purification of any such factors from the extracellular medium, human peripheral blood mononuclear cells (PBMCs) were cultured in serum-free medium. Some of the cells became long, spindle-shaped cells after 3–5 days (81, 82). Videomicroscopy indicated that these spindle shaped cells were quite motile, and they stained for fibrocyte markers (81). Fibrocytes did not appear during this timeframe when serum was present, indicating that something in serum inhibits fibrocyte differentiation.

Since removing something that inhibits fibrocyte differentiation might potentiate wound healing, and conversely adding something that inhibits fibrocyte differentiation might inhibit fibrosis, we abandoned the search for human density sensing factors and purified the fibrocyte differentiation inhibitor from human serum. It turned out to be SAP (81). SAP also inhibits the differentiation of mouse, rat, and dog PBMCs into fibrocytes (83–86). CRP has no significant effect on, and PTX3 potentiates, fibrocyte differentiation, indicating that the three pentraxins differentially affect fibrocytes (81, 87). When PBMCs were cultured in serum that was depleted of SAP, fibrocytes rapidly appeared, indicating that SAP is the main endogenous inhibitor of fibrocyte differentiation in the blood (81).

Pentraxins also regulate macrophages (17, 39, 88–95). In addition to inhibiting fibrocyte differentiation, SAP inhibits pro-fibrotic macrophages, and promotes the formation of immuno-regulatory macrophages (84, 95–105). Although SAP can bind complement component C1q and mannose-binding lectin, these proteins have very modest effects on the ability of SAP to affect macrophage phenotypes (95, 106–108). We refer the reader to the above references and reviews for information on the complexity of pentraxin (including SAP) regulation of macrophages that is beyond the simplicity of this minireview (Figure 1).

## Removing SAP as a possible therapeutic for wound healing

Since after blood clots, a wound is covered with serum, and serum contains SAP, and SAP inhibits fibrocyte differentiation and thus wound healing, an intriguing possibility is that removing SAP from wound fluid might potentiate fibrocyte differentiation and wound healing. Wound dressings with Ca^++^ and the type of agarose originally used to purify SAP from serum were tested on full thickness dermal wounds in rats. These dressings speeded healing of these wounds, as well as partial thickness dermal wounds in pigs (109). In the pig wounds, the agarose/ Ca^++^ dressings caused wound to heal faster than wounds treated with commercial dressings such as Tielle, Intrasite, and Xeroform. Although SAP levels in humans are unaffected by inflammation, serum SAP levels in the general population range from 20 to 60 μg/ml (3, 5, 110). Compared to controls, patients with low levels of SAP have better survival of skin grafts, supporting the idea that reducing SAP levels might help wound healing (111). In part because the wound dressing market is basically saturated, efforts to fund clinical tests of this SAP-depleting dressing have been unsuccessful.

## Adding SAP as a possible therapeutic for fibrosing diseases

A simple non-surgical animal model of a fibrosing disease is pulmonary fibrosis in mice and rats, where a drug called bleomycin can be pipetted through the mouth into the airway, and within 14 days causes pulmonary fibrosis (112, 113). In the bleomycin model, SAP injections led to reduced numbers of fibrocytes in the lungs and reduced fibrosis in rats and mice, and delaying SAP injections until inflammation and fibrosis was already apparent (therapeutic dosing) also reduced symptoms (84). SAP injections have now been shown to inhibit inflammation and fibrosis in other models of pulmonary fibrosis (102, 103), cardiac fibrosis (96, 97), radiation-induced oral mucositis (101), allergic airway disease (100), autoimmune encephalomyelitis (114), corneal wound healing (75), and two models of renal fibrosis/ end stage kidney disease (98). An obvious question about using SAP as a therapeutic for fibrosis is that this might block wound healing. We found that SAP injections slow, but do not stop, dermal wound healing in mice (115).

## SAP efficacy as an antifibrotic in clinical trials

Compared to control mice, mice lacking SAP have strongly increased pulmonary fibrosis in response to bleomycin, indicating that an endogenous function of SAP is to reduce fibrosis (116). Compared to controls, patients with renal fibrosis, pulmonary fibrosis, scleroderma, myelofibrosis, rheumatoid arthritis, and mixed connective tissue disease tend to have low levels of SAP, supporting the idea that fibrosis might in part involve a SAP deficiency (81, 98, 103, 117). One initial problem obtaining NIH funding to study SAP and fibrosis was that people confused SAP with serum amyloid A (SAA; a completely different, and probably not beneficial protein). We encountered this with a grant application where a reviewer denounced our efforts to inject animals with SAA. After politely explaining that SAP was not SAA, the grant was funded. An early worker in the SAP field encountered this too, and published a letter in Nature entitled “Serum Amyloid P component (not Serum Amyloid Protein)” (118). For this and other reasons, colleagues used the alternative SAP nomenclature Pentraxin 2 (PTX2) (26, 119), and called the recombinant SAP used for clinical trials PRM-151 (120). Compared to standard of care, injections of recombinant human SAP/PTX2 improved lung function in a Phase 1b trial and a Phase 2 trial in pulmonary fibrosis patients (5, 121). In the 28-weeks Phase 2 trial, SAP injections slowed the decline in forced exhalation volume (FEV), and essentially stopped the decline in the distance patients could walk in 6 min. 61 of the patients receiving SAP in this trial were also taking either pirfenidone or nintedanib, FDA-approved drugs that slow the decline in lung function in pulmonary fibrosis, while 16 other patients treated with SAP were not taking these drugs. Intriguingly, the 16 patients not taking these drugs who were treated with SAP appeared to show on average a very slight improvement in forced exhalation volume and an improvement in how far they could walk in 6 min, suggesting the exciting possibility that SAP might be able to partially reverse pulmonary fibrosis. Myelofibrosis is a fibrosis of the bone marrow (122). SAP injections also reduced fibrosis and improved bone marrow function in a 27-patient Phase 2 trial on myelofibrosis patients (123).

## SAP pharmacology

The plasma clearance rates for patients treated with SAP is ~24 to 30 h (5, 124). In the Phase 2 clinical trial, and in earlier trials, efficacy was observed with monthly dosing. The apparent paradox of how something with a short plasma half-life could show efficacy with monthly dosing can be answered by looking at the tissue half-life, which for healthy volunteers is 7.2 days (124). SAP, as well as CRP and PTX3, have long been known as opsonins that helps phagocytic cells ingest debris (16, 125–129). In fibrosis, debris and other tissue insults are thought to both initiate as well as potentiate fibrosis (39). Clearing debris from the vicinity of a fibrotic lesion is very likely one mechanism whereby SAP inhibits fibrosis. The debris is not detectable in the circulation, rather it is localized to the vicinity of the fibrotic lesion. Thus this beneficial effect of SAP occurs in the tissue rather than in the circulation. Although SAP has some modest effects on macrophage differentiation from monocytes (changes in the expression of a small number of surface markers in some but not all of the macrophages) (99, 95), macrophage polarization from one macrophage phenotype to another macrophage phenotype (again, even more subtle changes in the expression of a small number of surface markers) (95), and neutrophil adhesion to tissue extracellular matrix components (see below), the most obvious effect of SAP on innate immune cells is its ability to completely inhibit the differentiation of monocytes into fibrocytes. All of these effects on innate immune cells affect what the cells do after they have entered a tissue. A reasonable assumption is thus that the SAP effects occur in the tissue, specifically in the vicinity of the fibrotic lesion, rather than in the circulation, and thus that the key half-life is the tissue rather than plasma half-life.

Two observations suggest that the half-life of SAP in a fibrotic lesion may be considerably longer than 7.2 days. First, amyloid deposits resemble in many ways fibrotic lesions, and the half-life of SAP in amyloid deposits is 24–27 days (124, 130, 131). Second, in mice where fibrosis was induced in one kidney by obstructing the ureter, injected SAP localized to the fibrotic kidney, with much less localization to the non-injured contralateral kidney (98). Together, these arguments and results support the idea that even with a short plasma half-life, monthly injections of SAP can be efficacious.

## SAP inhibition of fibrocytes allows an assessment of the possible effect of factors such as dietary salt on fibrosis

Human PBMC cultured in serum-free medium differentiate into easily identifiable (by microscopy) fibrocytes, and adding different concentrations of SAP to inhibit this generates a standard curve of SAP effects. This allows a simple assay to look at the effects of various conditions or compounds on this process. For instance, ELISA assays of sera from keloid patients (these patients form greatly exaggerated dermal scars) showed normal levels of SAP, but the fibrocyte assay on keloid patient PBMC showed that these cells are relatively insensitive to SAP (132). A variety of compounds affect fibrocyte differentiation and/or the ability of SAP to inhibit fibrocyte differentiation (57, 63, 82, 87, 117, 133–142). One compound that may be clinically relevant is NaCl, which when added to increase the medium NaCl concentration by 25 mM (this level of increase can be seen in the serum after a very salty meal) potentiates fibrocyte differentiation and inhibits the SAP effect, possibly explaining why high salt intake is associated with a propensity for cardiac fibrosis (137). Peritoneal dialysis can lead to peritoneal fibrosis, and we found that peritoneal dialysis fluid and dialysis fluid components such as NaCl also promote fibrocyte differentiation and impede SAP (139). In support of this connection between salt and fibrocytes, low dietary salt reduces the severity of bleomycin-induced pulmonary fibrosis in mice, suggesting that low salt diets may be beneficial for fibrosis patients (143).

## SAP can override other profibrotic factors

A variety of signals promote wound healing and fibrosis. For instance, TGF-β1 is an extracellular signal that drives fibrosis (144, 145), and in mice, conditional expression of TGF-β1 in the lungs causes pulmonary fibrosis (103, 146). In this model, SAP injections stopped and reversed fibrosis (103). We found that although quiescent fibroblasts secrete the protein Slit2 to inhibit fibrocyte differentiation (essentially telling incoming monocytes that no more fibroblast-cells are needed) (138), fibroblasts activated by the pro-fibrotic signal TNF-α secrete the protein lumican, which promotes fibrocyte differentiation (142). Thankfully, SAP can override the effect of lumican on fibrocytes (142). Other signals that promote fibrocyte differentiation and profibrotic macrophage differentiation include thrombin activated during blood clotting (this may thus initiate the fibrocyte component of wound healing), and tryptase released from mast cells (147, 148). SAP also competes with these signals to inhibit fibrocytes and macrophages (149, 148). In addition, SAP inhibits fibrocyte differentiation induced by IL-4, IL-13, high molecular weight hyaluronic acid, and PTX3 (82, 87, 136, 141). Together, these results suggest that one reason SAP appears to be effective in the clinic as an anti-fibrotic is a fortunate dominance of SAP over these signals.

## Elucidating SAP receptors led to small-molecule SAP mimetics

Fcγ receptors (FcγRs) bind the Fc domain of IgG immunoglobulins (150). Once aggregated IgG cross-links multiple FcγRs (this prevents monomeric IgGs from activating FcγRs), a signaling cascade is activated through tyrosine kinases to initiate an immune response (151). Phagocytic cells, such as monocytes, bind SAP, CRP, and PTX3 using different combinations of FcγRs (16, 86, 98, 152–156), and the structure of SAP bound to FcγRIIa and modeling of SAP binding to other Fc receptors has been published (157, 158). In support of the hypothesis that SAP inhibits fibrocyte differentiation by binding to FcγRs, we found that cross-linked but not monomeric IgG also inhibits fibrocyte differentiation (159). Mouse monocytes lacking FcγRI, or human monocytes with siRNA-reduced FcγRI, had a reduced sensitivity to SAP, while mouse cells lacking other FcγRs had normal or enhances sensitivity to SAP, indicating that FcγRI mediates SAP signaling (86). Surprisingly, monocytes from cells lacking all four known FcγRs still responded to SAP, indicating that a different receptor also mediates SAP signaling (156, 160).

To help elucidate SAP signaling, we mutated SAP protein surface amino acids that were different from CRP, and the mutant SAPs were assayed for their ability to inhibit fibrocyte differentiation (SAP and CRP are have highly similar amino acid sequences and structures, but CRP does not inhibit fibrocyte differentiation) (81, 87)). None of the mutant SAPs completely abrogated SAP activity (86, 156). One amino acid initially overlooked was a glycosylated asparagine on SAP that is a non-glycosylated alanine on CRP, and when SAP was desialylated, the SAP largely lost its ability to inhibit fibrocyte differentiation; conversely when the CRP alanine was mutated to an asparagine, the asparagine became glycosylated and the glycosylated CRP inhibited fibrocyte differentiation (160). This suggested that a polysaccharide receptor might help to sense SAP, and we found that the C-type lectin DC-SIGN mediated SAP effects on monocytes (160). Other workers found a variety of compounds that block the ability of polysaccharides to bind DC-SIGN, and three of these potently inhibited fibrocyte differentiation. One of the DC-SIGN-binding molecules showed efficacy in a mouse pulmonary fibrosis model at 0.001 mg/kg (160). These results suggest that small molecules that mimic SAP might be useful as therapeutics for fibrosing diseases.

## Adding SAP as a possible therapeutic for neutrophil-driven diseases

Inflammatory lesions recruit neutrophils to the site of damage (161, 162). This however can sometimes be counterproductive; for instance some patients with damaged lungs develop acute respiratory distress syndrome/ acute lung injury (ARDS/ ALI), where neutrophils enter the lungs and release proteases and reactive oxygen species. This causes further damage and further neutrophil recruitment and subsequent damage, and this vicious cycle results in the ~40% mortality seen in the ~200,000 ARDS patients each year in the US (163). SAP decreases neutrophil binding to extracellular matrix components (164–166), and in a mouse model of ARDS, SAP injections starting 24 h after injury reduced the number of neutrophils in the lungs (166). The small-molecule SAP mimetic discussed above also showed efficacy in this ARDS model (160). These results suggest that SAP and SAP mimetics might be useful as therapeutics for neutrophil-driven diseases such as ARDS/ ALI.

## Blocking SAP signaling as a possible therapeutic for diseases such as tuberculosis

M1 macrophages are highly aggressive against bacteria and other pathogens, but SAP, which is a constitutive component of the blood, pushes macrophages toward an anti-inflammatory/anti-fibrotic phenotype (90, 95, 99, 100, 103, 156, 167, 168). Tuberculosis bacteria can live inside macrophages, where they also push the host macrophage away from a M1 phenotype to help the survival of the parasitic bacteria (169). To test the hypothesis that blocking SAP signaling to macrophages would reduce regulatory macrophages and increase M1 macrophages, we screened 3,000 compounds for the ability to inhibit the binding of SAP to FcγRI, and found 12 that reduced this binding (170). In support of the hypothesis, SAP potentiated the proliferation of *Mycobacterium smegmatis* and *Mycobacterium tuberculosis* in human macrophages, and in the presence of SAP, 2 of the compounds reduced the intra-macrophage proliferation of these bacteria (170).

## Conclusion

Pentraxins are ancient and fascinating molecules. Increasing levels of SAP either locally or systemically is showing promise as a therapeutic for a variety of diseases where the ability of SAP to help clear debris and calm the innate immune system is beneficial. Conversely, decreasing levels of SAP, or decreasing SAP effects, shows promise as potential therapeutics where unleashing the innate immune system is beneficial. An intriguing possibility is that altering levels of other pentraxins might similarly be useful as stand-alone therapeutics or in combination with manipulations of SAP levels for even more diseases.

## Author contributions

All authors listed have made a substantial, direct and intellectual contribution to the work, and approved it for publication.

### Conflict of interest statement

DP and RG are inventors on patents for the use of SAP as a therapeutic for fibrosing diseases, and patents for the use of SAP-depleting materials to enhance wound healing. DP and RG are members of the Science Advisory Board of, and have stock options from, Promedior, a start-up company that is developing SAP as a therapeutic for fibrosing diseases, and receive a share of milestone payments made by Promedior to Rice University. The reviewer AM and handling Editor declared their shared affiliation.
